# Validity of sagittal thoracolumbar curvature measurement using a non-radiographic surface topography method

**DOI:** 10.1007/s43390-022-00538-0

**Published:** 2022-07-09

**Authors:** Erin Hannink, Helen Dawes, Thomas M. L. Shannon, Karen L. Barker

**Affiliations:** 1 Physiotherapy Research Unit, Nuffield Orthopaedic Centre, Oxford University Hospitals NHS Foundation Trust, Oxford, UK; 2 Centre for Movement, Occupational and Rehabilitation Sciences, Oxford Brookes University, Oxford, UK; 3 Nuffield Department of Orthopaedic, Rheumatoid and Musculoskeletal Sciences, University of Oxford, Oxford, UK; 4 College of Medicine and Health, University of Exeter, Exeter, UK; 5 Oxford Health, Biomedical Research Centre, University of Oxford, Oxford, UK; 6 Centre for Biomechanics and Rehabilitation Technologies, Staffordshire University, Stoke-on-Trent, UK

**Keywords:** Kyphosis, Lordosis, Spine, Validity

## Abstract

**Purpose:**

To estimate the criterion validity of sagittal thoracolumbar spine measurement using a surface topography method in a clinical population against the gold standard and to estimate concurrent validity against two non-radiographic clinical tools.

**Methods:**

In this cross-sectional validity study, thoracolumbar curvature was measured in adults with spinal conditions recruited from a specialist orthopaedic hospital. A surface topography method using a Kinect sensor was compared to three other measurement methods: spinal radiograph (gold standard), flexicurve and digital inclinometer. Correlation coefficients and agreement between the measurement tools were analysed.

**Results:**

Twenty-nine participants (79% female) were included in criterion validity analyses and 38 (76% female) in concurrent validity analyses. The surface topography method was moderately correlated with the radiograph (*r* = .70, *p* < .001) in the thoracic spine, yet there was no significant correlation with the radiograph in the lumbar spine (*r* = .32, *p* = .89). The surface topography method was highly correlated with the flexicurve (*r*_s_ = .91, *p* < .001) and digital inclinometer (*r* = .82, *p* < .001) in the thoracic spine, and highly correlated with the flexicurve (*r* = .74, *p* < .001) and digital inclinometer (*r* = .74, *p* < .001) in the lumbar spine.

**Conclusions:**

The surface topography method showed moderate correlation and agreement in thoracic spine with the radiograph (criterion validity) and high correlation with the flexicurve and digital inclinometer (concurrent validity). Compared with other non-radiographic tools, this surface topography method displayed similar criterion validity for kyphosis curvature measurement.

## Introduction

Abnormal sagittal spinal curvature is associated with poor health-related outcomes such as pain, decreased mobility, respiratory problems, and increased mortality [1–3]. While a thoracic kyphosis angle of 20°–40° is generally accepted as the normal range, curvature changes can progress both with age and due to certain spinal conditions, resulting in excessive kyphosis (hyperkyphosis) in approximately one third of older adults [2]. For example, older women have been shown to have an increased thoracic kyphosis angle of 7° over 15 years, and furthermore each osteoporotic vertebral fracture can increase the kyphosis angle 3°–4° [4]. Additionally, changes over time in the lumbar lordosis curvature can cause hyperlordosis, due to compensatory mechanisms or conditions such as spondylolisthesis, or hypolordosis, a flattening of the lumbar spine often linked to degenerative disc disease [5–7]. Therefore, the ability to measure thoracolumbar sagittal curvature is crucial.

A surface topography method using the Microsoft Kinect sensor V2 (Microsoft Corporation, Seattle, Washington, U.S.A) has been developed to measure spinal curvature. It is an extremely adaptable commercial device that has been employed in many different applications in the healthcare environment [8]. Utilising time-of-flight technology, a 3D image of the back can be quickly and economically reconstructed using the phase shift of an infrared beam to create a large cloud of pixels, each with an encoded distance to digitally describe the surface of an object [8–10]. Within the realm of posture and movement research, the Kinect sensor has been tested in multiple capacities, from movement analysis to postural control and ergonomic positions to the cosmetic defect of the back surface [11–16].

Two studies have tested reliability and validity of the surface topography method using the Kinect sensor in healthy volunteers [16, 17], yet there is a need to test this method in a clinical cohort. To test the criterion validity, the current gold standard is the Cobb angle from a lateral view spinal radiograph which produces an angle derived by vertebral body alignment [18, 19]. While this method is the gold standard, its drawbacks include the operating and instrument costs, and most importantly, the exposure to ionising radiation [18, 20]. Therefore, it is also important to test the concurrent validity using the flexicurve and the digital inclinometer which are established in research and clinical use [21, 22]. The primary aim of this study was to estimate the criterion validity of thoracolumbar sagittal spine measurement using a surface topography method compared to the radiograph; the secondary aims were to estimate the concurrent validity of the surface topography method compared to the flexicurve and digital inclinometer.

## Materials and methods

### Study design

This study was designed to assess criterion validity using the gold standard and concurrent validity using two surface measurement tools. It was conducted and evaluated according to the COnsensus-based Standards for the selection of health Measurement Instruments (COSMIN) pathway for validity [23]. We hypothesised that the surface topography method would demonstrate at least moderate validity and agreement with the radiographic and non-radiographic methods. The sample size was calculated for thoracic kyphosis measurement since there are limited lumbar lordosis data to reference; assuming the effect size (*r*^2^ = 0.57) obtained from the criterion validity of the flexicurve [24] and based on the strong correlation found between the surface topography method and flexicurve (ICC = 0.77) [16], with an alpha error of 0.05 and power 0.80, a sample size of 26 participants was required. The study had ethical approval by the South West—Central Bristol Research Ethics Committee and it was conducted in compliance with the Declaration of Helsinki.

### Participants

Men and women over 18 years old were recruited from a specialist orthopaedic hospital and informed consent was obtained from all participants. Participants were included for concurrent validity if they were attending the hospital for a spinal condition. If they had undergone a recent spinal radiograph, they were eligible for criterion validity analysis. Exclusion criteria included inability to stand independently or a diagnosed neurological condition affecting trunk control. If a participant reported any change in functional status or had undergone a medical procedure or treatment between the time of the radiograph and the research visit, or if the spinal radiograph could not be accurately measured due to poor image quality or obstructive spinal implants, they were excluded from criterion validity analysis.

### Procedures

**Fig. 1:**
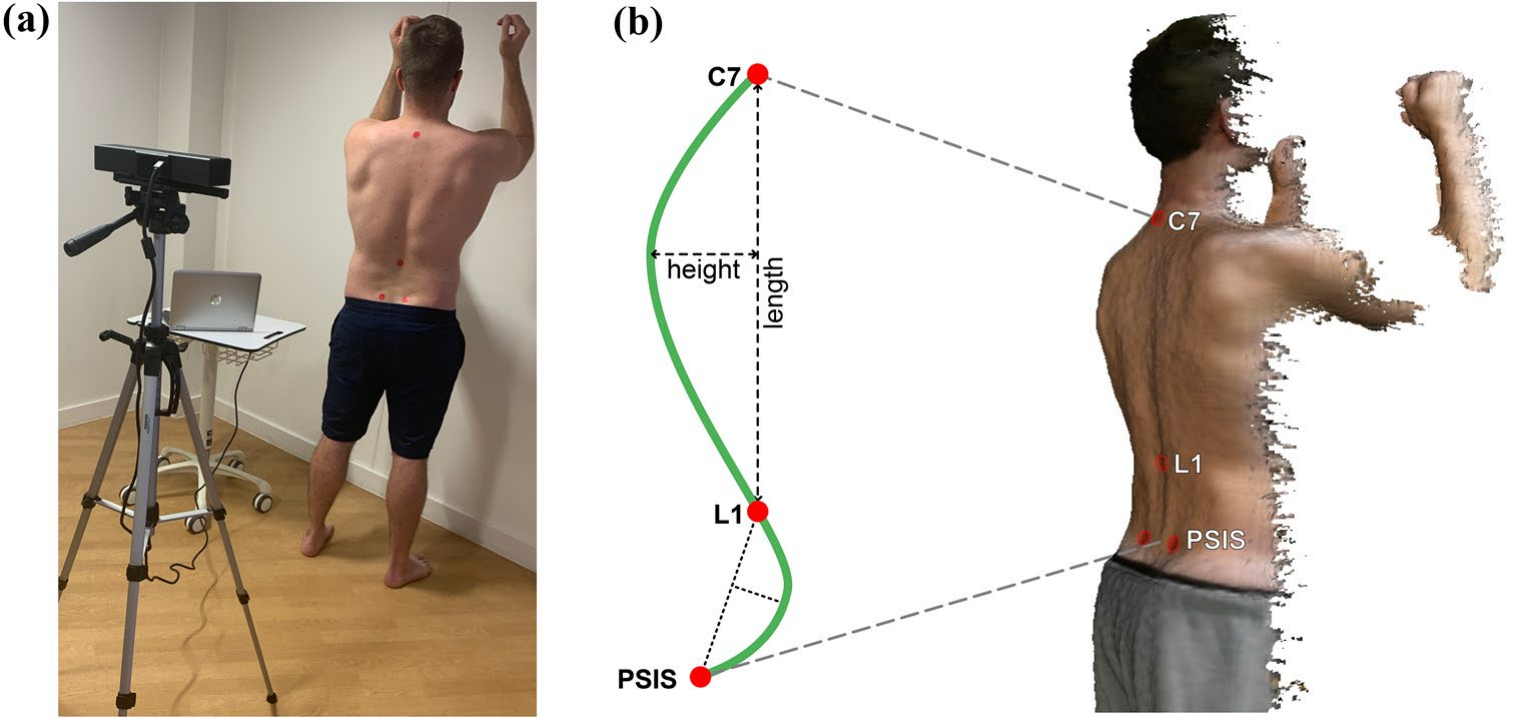
**a** An example of the surface topography set-up using the Kinect sensor, **b** the reconstructed surface topography image with the anatomical landmarks used for the non-radiographic measurement methods and the schematic depicting kyphosis and lordosis indexes

The primary study outcomes were thoracic and lumbar curvature measured by the surface topography method compared to the radiographic Cobb angles. The secondary outcomes included the measurements obtained from the flexicurve and digital inclinometer. During the research visit at an orthopaedic hospital, a single assessor, with more than 5 years of musculoskeletal physiotherapy experience, measured each participant using the three non-radiographic tools. The assessor palpated the participants’ spine and placed adhesive markers to identify anatomical landmarks: C7, L1, and right and left posterior superior iliac spine (PSIS). To decrease variability a standardised protocol for landmark identification was implemented; while surface palpation of spinal processes has limited validity compared to radiograph, identification has shown acceptable reliability, with inaccuracies impacting frontal plane curvature more than sagittal plane [25, 26]. Assessor instructions for all tools were standardised for participants to stand in their ‘best posture’ with their shoulders and elbows in 90° of flexion (Fig. 1) to correspond with instructions given during the spinal radiograph. The order of measurement for the three non-radiographic tools was randomly selected before the research visit.

#### Surface topography method

The surface topography method using the Kinect sensor in healthy populations has demonstrated good concurrent validity against the flexicurve (ICC = 0.77), very high intrarater (ICC = 0.96–0.98) and interrater reliability (ICC = 0.97) for thoracic kyphosis measurement, and very high intrarater (ICC = 0.97–0.98) and interrater reliability (ICC = 0.97) for lumbar lordosis measurement [16, 17]. Procedural methods and data processing for the surface topography method were based on a previous reliability study [17]. The Kinect sensor, mounted on a tripod, was adjusted in height to be level with the participants’ mid-scapular region and participants stood with their heels at a 1-metre distance facing away from the sensor (Fig. 1). The assessor was blinded to the curvature measurements as they were processed after the research visit. Angle indexes were calculated using the length of the target spinal region and the maximum height, an accepted, validated method used with the flexicurve [24], e.g., kyphosis index = [height/(length from C7 to L1)] × 100. The angle index calculation was applied to the lumbar index with the length extending from L1 to bisection of right and left PSIS (Fig. 1).

#### Radiographic Cobb angle

For participants with a standing lateral view spinal radiograph, the digital radiographic image was measured with Cobb angles. The thoracic kyphosis angle was digitally generated from the intersection of a line parallel to the superior vertebral endplate of T4 and inferior endplate of T12, with T4 is used instead of T1 to avoid the commonly obstructed view of the upper thoracic vertebrae [19]; the lumbar lordosis angle was generated from the superior endplate of L1 and inferior endplate of L5 [18, 27].

#### Flexicurve

The flexicurve is a flexible ruler moulded along the spine (C7 to S2) and traced onto graph paper; it has demonstrated high reliability with mixed validity for thoracic kyphosis and lumbar lordosis measurement [21, 22, 28]. The kyphosis index and lordosis index were derived using the same method from the surface topography procedures. For conversion to kyphosis angle from the kyphosis index, the conversion equation from Greendale et al. [24] was used: Kyphosis angle = (3.1461 × kyphosis index) + 5.11.

#### Digital inclinometer

The digital inclinometer (The Saunders Group, Inc, Chaska, MN, U.S.A.) is an instrument that measures the relative angle between two inclinometers with respect to gravity; it has demonstrated high reliability and moderate validity in thoracic kyphosis measurement and moderate to high reliability with mixed validity in lumbar lordosis measurement [21, 22, 28, 29]. The thoracic angle was measured by placing one unit at C7 and the second unit at L1; the lumbar angle was measured using L1 and the bisection of PSIS.

### Statistical analysis

Descriptive and frequency statistics of participants and curvature characteristics were analysed and reported. Preliminary analyses tested the normality of each variable using the Shapiro–Wilk test. Correlation analysis was based on Pearson’s correlation coefficients for parametric variables and Spearman’s Rank-order correlation for non-parametric variables. Correlation coefficients were interpreted as very high (0.90–1.00), high (0.70–0.90), moderate (0.50–0.70), low (0.30–0.50), and negligible (0.00–0.30) [30]. A simple linear regression analysis for the radiographic Cobb angle was used to create a conversion equation for the surface topography kyphosis index. Agreement between measurement values was assessed using Bland–Altman plots and 95% confidence intervals. Statistical significance determined by *p* < 0.05. Data were analysed using SPSS software (IBM SPSS Inc version 25, Chicago, IL).

## Results

Thirty-eight participants were recruited to the concurrent validity study and 29 of these participants had a radiograph eligible for primary outcome criterion validity analysis. Of the full sample, the mean age was 58.8 (SD 16.9) years old and body mass index (BMI) was 24.9 (SD 4.2) kg/m^2^; 76% were female and 61% reported back pain with a mean of 4.6 (SD 2.6) on the pain visual analogue scale (0–10, 10 representing the highest level of pain) (Table 1). Participants had a diverse range of primary spinal conditions including osteoporosis (*n* = 4), ankylosing spondylitis (*n* = 4), low back pain with and without radiating symptoms (*n* = 8), vertebral fracture (*n* = 2), mild to moderate scoliosis (*n* = 17), and spinal stenosis (*n* = 3); five participants had previous lumbar spinal fusion surgery (*n* = 2 single level, *n* = 3 multilevel). Descriptive measurements of the spinal curvature display an array of both thoracic and lumbar curves, spanning beyond normal ranges of kyphosis and lordosis (Table 2). The mean time span between the radiograph and the research visits was 49 days. There were no missing data.

**Table 1:** Demographic and physical participant characteristics

Full sample, *n* = 38	
Age, y [mean ± SD (range)]	58.7 ± 16.9 (22–82)
BMI, kg/m^2^ [mean ± SD (range)]	24.9 ± 4.2 (19.1–37.6)
Gender [female *n* (%)]	29 (76%)
Pain [symptomatic *n* (%)]	23 (60.5%)

Radiograph subgroup, *n* = 29	
Age, y [mean ± SD (range)]	56.9 ± 18.2 (22–82)
BMI, kg/m^2^ [mean ± SD (range)]	24.7 ± 4.3 (19.1–37.6)
Gender [female *n* (%)]	23 (79%)
Pain [symptomatic *n* (%)]	20 (70%)

**Table 2:** Descriptive thoracolumbar curvature characteristics

	Mean (SD)	Range
Radiograph, *n* = 29		
Thoracic kyphosis angle (°)	43.8 (14.0)	19.4–88.3
Lumbar lordosis angle (°)	40.9 (16.5)	14.8–83.1
Surface topography, radiograph subgroup, *n* = 29		
Kyphosis index	13.4 (4.5)	6.7–25.2
Lordosis index	8.6 (4.0)	1.3–16.1
Surface topography, full sample, *n* = 38		
Kyphosis index	14.3 (5.7)	5.5–30.4
Lordosis index	8.6 (4.0)	1.3–16.1

### Thoracic spine

**Fig. 2:**
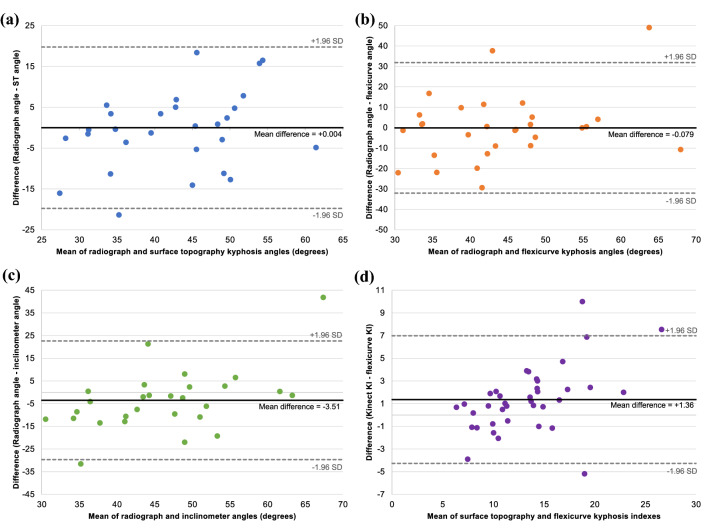
Bland–Altman plots for the thoracic region include the mean difference and 95% CI as the upper and lower limits; the charts display the agreement between **a** radiograph and surface topography angles, **b** radiograph and flexicurve angles, **c** radiograph and digital inclinometer angles, and **d** the surface topography kyphosis index and flexicurve kyphosis index

In the analysis of criterion validity, surface topography demonstrated moderate correlation with the radiograph (*r* = 0.70, *p* ≤ 0.001) and high correlation with the digital inclinometer (*r *= 0.82, *p* ≤ 0.001) and flexicurve (*r*_s_ = 0.91, *p* ≤ 0.001) (Table 3). A linear regression analysis of thoracic angle using the surface topography kyphosis index values produced a conversion equation (adjusted *R*^2^ = 0.47): Thoracic angle = (2.16 × kyphosis index) + 15.05. Using this conversion equation, the agreement between surface topography and radiograph showed minimal positive bias towards the radiographic angle with one outlier beyond the lower limit of agreement and normal distribution of differences (*p* = 0.45) (Fig. 2). For comparison, agreement between the flexicurve and digital inclinometer with the radiograph show two outliers beyond the limits of agreement. The agreement between surface topography and flexicurve show more dispersion and less agreement with three outliers as the angle indexes increase.

**Table 3:** Correlation matrix for all measurement methods of the thoracic spine and lumbar spine

	Radiograph (*n* = 29)	Flexicurve (*n* = 38)	Digital inclinometer (*n* = 38)
Thoracic spine			
Surface topography	.70*	.91*^,a^	.82*
Digital inclinometer	.67*	.76*^,a^	
Flexicurve	.54*^,a^		
Lumbar spine			
Surface topography	.32	.74*	.74*
Digital inclinometer	.34	.77*	
Flexicurve	.28		

*p < .05

^a^Spearman’s rank-order correlation coefficient

### Lumbar spine

Lumbar spine curvature showed high correlation between surface topography and the flexicurve and digital inclinometer (*r* = 0.74, *p* ≤ 0.001 and *r* = 0.74, *p* ≤ 0.001, respectively) (Table 3). There was no correlation between the radiograph and surface topography (*r* = 0.32, *p* = 0.89) and similarly no significant correlation between the radiograph and the flexicurve or digital inclinometer. Since a significant linear regression was not available to convert the surface topography lordosis index to an angle, agreement could not be analysed.

## Discussion

The surface topography method demonstrated moderate correlation and good agreement with radiographic measures in the thoracic spine; however, lumbar lordosis did not correlate with the radiographic measures. The surface topography method showed good concurrent validity as demonstrated by the strong correlation with the flexicurve and digital inclinometer in both the thoracic and lumbar regions.

### Thoracic spine

The validity results in the thoracic spine demonstrate a consistently high correlation between all measurement tools. The Bland–Altman plots showed agreement between methods apart from a few outliers. Not only does the surface topography method correlate and agree with radiographic measurement, the flexicurve and digital inclinometer do as well. The flexicurve and surface topography likely have tighter correlations because they share a very similar method of measuring curvature and computation of an angle index. The highly correlative relationship between surface topography and flexicurve is consistent with the results from Quek et al. who found strong correlation between these methods (ICC = 0.77) in a healthy population [16]. Since the relationship between the flexicurve and digital inclinometer with the radiograph (Table 3) are comparable to previous studies [24, 29, 31], they serve as a good reference for the performance of the surface topography method, which demonstrated similar correlation and agreement with the radiograph. When comparing the criterion validity of the surface topography method to other technologies, the Kinect sensor (*r* = 0.70) demonstrates a similar correlation to the radiograph as another surface topography method, rasterstereography (*r* = 0.75) [32]. These results suggest that the surface topography method employing the Kinect sensor performs comparably to other established non-radiographic tools for thoracic kyphosis measurement.

### Lumbar spine

Contrary to thoracic spine findings, there was no significant correlation between surface topography and radiographic measurements in the lumbar spine. There was however a strong correlation between the surface topography method and the flexicurve and digital inclinometer (Table 3), which could be attributed to the surface measurement, as opposed to vertebral body alignment. The lack of a correlative relationship with the radiograph could be rationalised by the increased soft tissue overlaying the spinous processes specific to the lumbar region, and this is further influenced by BMI, body morphology and the angle of lumbar lordosis [25, 33, 34]. These factors help explain the poor and inconsistent correlation with radiographic measures demonstrated in this study as well as other studies in the literature [21]. Of all the non-radiographic methods, rasterstereography has demonstrated the strongest correlation with the radiograph (*r* = 0.71), yet six of the seven studies in the meta-analysis investigated adolescent idiopathic scoliosis (AIS) which is a population with lower BMI on average [32]. Conversely, in a degenerative disc disease cohort, the lumbar lordosis correlation with radiography was weak [32, 35]. In addition, Applebaum et al. recently reported significant differences between rasterstereography and radiography measurement [36], which aligns with our findings and further demonstrates the difficulties of lumbar surface measurement.

### Utility of the Kinect sensor for surface topography

Compared to the flexicurve and digital inclinometer, surface topography using the Kinect sensor is more equipment-intensive, and although it consumes the same amount of patient-facing time, it requires a few more minutes to set-up and to gather output after. These extra resources must be balanced by the added value, as it can provide a more robust curvature profile without additional technical or clinical expertise. Surface topography methods have the capacity to estimate the transverse and frontal planes in addition to the sagittal plane, and they produce a topographical visualisation of the back surface potentially useful visual feedback for the patient. Measuring rotational curvature in an AIS population using the Kinect sensor has been shown to be comparable with radiographic measures [37]. While other surface topography tools, such as rasterstereography, also have the potential to measure multiple anatomical planes, most systems lack portability and affordability which are favourable attributes of the Kinect sensor [32]. Therefore, since the Kinect sensor demonstrates similar validity in the thoracic spine, in a cohort where rotational deformity is present or where visual feedback would be important, this method could be clinically valuable and offer output that the flexicurve and digital inclinometer cannot. When investigating surface topography in reference to radiography, there is complementary role for its clinical utility. The cumulative effective dose from long-term ionising radiation exposure incurred by repetitive spinal radiographs has demonstrated an increased association with cancer risk; while the risk is small, surface topography would be useful to monitor change in the interim of radiographs in progressive spinal conditions [38, 39]. Three-dimensional postural monitoring would also be beneficial when a spinal radiograph is not clinically indicated for the patient. Surface topography definitively cannot replace spinal radiography, but it does have the potential to be an adjunct measurement to monitor change in spinal curvature and posture. Although the Kinect sensor V2 used in this study is no longer commercially available, the Azure Kinect is the next generation designed to target research utility with the focal upgrade being its ability to utilise an artificial intelligence cloud platform. The underlying time-of-flight technology did not change thus allowing the methods of image and data analysis from this study to remain relevant.

### Limitations

The limitations in this study include the sample size, which was powered for validity analysis of the thoracic spine, yet not powered for the lumbar spine. While the clinical population had a variety of spinal curvature profiles, there were too few with severe kyphosis to fully generalise results into the hyperkyphotic population and the sample may have been too heterogeneous to detect a correlation with the radiograph in the lumbar spine. We would hypothesise that higher BMI levels would negatively affect validity in the lumbar region as BMI is one of the factors associated with increased soft tissue depth along the lumbar spinous processes [33, 34]. There is also a possibility that scoliosis influences curvature measurement, however Severjins et al. has shown scoliosis leads to medial–lateral error that primarily affects coronal measurement, and in addition error corresponds with more severe rotational curvature [25]. It would be useful for a future study increase sample size to take into account these possible confounding variables. In addition, non-radiographic measurements were not conducted concurrently with the radiographs; since functional changes of status and medical procedures were screened, we do not expect that sagittal spine alignment changed more than normal postural variation between measurements, however this remains a limitation of the study design.

## Conclusions

The study findings suggest that the surface topography method using the Kinect sensor is a valid tool to measure thoracic kyphosis when compared to both the accepted gold standard (radiograph) and clinically used non-radiographic tools (flexicurve and digital inclinometer). While the findings indicate that surface topography cannot substitute a radiograph, the method can be used to measure the thoracic curvature, which is a clinically important outcome for a clinician to track and monitor.
